# Identification of m6A methyltransferase-related lncRNA signature for predicting immunotherapy and prognosis in patients with hepatocellular carcinoma

**DOI:** 10.1042/BSR20210760

**Published:** 2021-06-08

**Authors:** Lili Li, Rongrong Xie, Guangrong Lu

**Affiliations:** 1 Department of Medical Oncology, The First Affiliated Hospital of Wenzhou Medical University, Wenzhou 325000, People’s Republic of China; 2 Department of Gastroenterology, The Second Affiliated Hospital and Yuying Children’s Hospital of Wenzhou Medical University, Wenzhou 325000, People’s Republic of China

**Keywords:** Hepatocellular carcinoma, Immune cells infiltration, Immune checkpoint, LncRNAs, N6-methyladenosine

## Abstract

N6-methyladenosine (m6A) methyltransferase has been shown to be an oncogene in a variety of cancers. Nevertheless, the relationship between the long non-coding RNAs (lncRNAs) and hepatocellular carcinoma (HCC) remains elusive. We integrated the gene expression data of 371 HCC and 50 normal tissues from The Cancer Genome Atlas (TCGA) database. Differentially expressed protein-coding genes (DE-PCGs)/lncRNAs (DE-lncRs) analysis and univariate regression and Kaplan–Meier (K–M) analysis were performed to identify m6A methyltransferase-related lncRNAs. Three prognostic lncRNAs were selected by univariate and LASSO Cox regression analyses to construct the m6A methyltransferase-related lncRNA signature. Multivariate Cox regression analyses illustrated that this signature was an independent prognostic factor for overall survival (OS) prediction. The Gene Set Enrichment Analysis (GSEA) suggested that the m6A methyltransferase-related lncRNAs were involved in the immune-related biological processes (BPs) and pathways. Besides, we discovered that the lncRNAs signature was correlated with the tumor microenvironment (TME) and the expression of critical immune checkpoints. Tumor Immune Dysfunction and Exclusion (TIDE) analysis revealed that the lncRNAs could predict the clinical response to immunotherapy. Our study had originated a prognostic signature for HCC based on the potential prognostic m6A methyltransferase-related lncRNAs. The present study had deepened the understanding of the TME status of HCC patients and laid a theoretical foundation for the choice of immunotherapy.

## Introduction

Worldwide, hepatocellular carcinoma (HCC) has become one of the most common malignancies and the second leading cause of cancer-related deaths [1]. HCC is the main pathological type of primary liver cancer, with a global incidence of 500000 new cases and more than 700000 deaths each year [2]. The major risk factors for HCC are chronic hepatitis B virus (HBV) and hepatitis C virus (HCV) infections, alcoholic liver disease, diabetes and non-alcoholic fatty liver disease [3]. The current curative treatments for early-stage HCC are surgery, thermal ablation, radiofrequency ablation or liver transplantation [4–7]. However, a large proportion of patients will have recurrence or distant metastasis after surgery [8]. For the more than 70% of patients diagnosed with advanced stage, treatments can only provide limited therapeutic benefit for a small subset of patients. Thus, elucidating the molecular mechanisms of HCC and identifying new molecular targets are essential for its diagnosis and treatment.

N6-methyladenosine (m6A), the most popular internal modification in eukaryotic messenger RNAs (mRNAs), microRNAs (miRNAs) and long non-coding RNAs (lncRNAs), plays a critical part in RNA splicing, stability, export and translation [9,10]. The dynamic modification of m6A is regulated by methyltransferases (m6A ‘writers’), demethylases (m6A ‘erasers’) and binding proteins (m6A ‘readers’) [11]. Numerous studies have described that m6A regulators are closely related to the occurrence and progression of a variety of cancers. It has been reported that m6A regulators are key participants in the malignant progression of gliomas and have potential roles in both prognosis and treatment strategy formulation [12]. Recently, many studies have revealed that abnormal m6A modification is involved in the development and progression of HCC [13–18]. *METTL14* inhibits the metastatic potential of HCC by regulating m6A-dependent primary miRNA processing [13]. Up-regulation of *YTHDF2* suppressed cell proliferation, tumor growth and activation of mitogen-activated protein kinase kinase (MAPKK/MEK) and extracellular signal-regulated kinase (ERK) in HCC cells [14]. KIAA1429 facilitated migration and invasion of HCC by altering m6A modification of *ID2* mRNA [16].

LncRNAs are families of non-coding molecular transcripts with over 200 nucleotides in length and have been recognized as having important functional roles in various human diseases [19,20]. The aberrant expression of lncRNAs is also closely related to the malignancy of tumors, and it is no exception in HCC [21–25]. For example, *lncRNA MIAT* was reported to promote proliferation and invasion of HCC cells via sponging *miR-214* [21]; up-regulation of *lncRNA SNHG16* inhibited HCC cell proliferation and chemotherapy resistance via functionally sponging *hsa-miR-93* [22] and *lncRNA HULC* can trigger autophagy by stabilizing Sirt1 and attenuate the sensitivity of HCC cells to chemotherapeutic agents [23]. However, the role of m6A regulators in the dysregulation of lncRNAs in cancer development is still unclear, and there are few studies focusing on the relationship between m6A modification and lncRNA-dependent HCC. Therefore, understanding the relationship between the modification of lncRNAs by m6A and the progression of HCC may contribute to identify biomarkers that may serve as therapeutic targets.

In the present study, we constructed an m6A-related lncRNA prognostic signature (*LINC02362, SNHG20* and *SNHG6*) which had a high accuracy in predicting overall survival (OS) and was an independent prognostic factor. Our results showed that this signature was implicated in immune-related terms and pathways that played a key role in HCC tumorigenesis, and was highly connected with the tumor microenvironment (TME) and immunotherapy responses. Our study had constructed a novel m6A-based prognostic model, which has potential prognostic value for patients with HCC and may be helpful for the guidance of personalized immunotherapy.

## Materials and methods

### Acquisition of gene expression and clinical data

Normalized RNA-sequencing data and the associated clinical information of the HCC samples were downloaded from the The Cancer Genome Atlas (TCGA) database. They included 374 tumor samples and 50 normal tissue samples. Three samples with metastasis were eliminated. Thus, our study included 371 tumor tissues. The normalized gene expression data of the TCGA-HCC database after log_2_-transformed were used for further analysis (**Supplementary Figure S1**). Samples from the TCGA database were divided randomly into a training set (260 tumor samples, 35 normal samples) and an internal validation set (111 tumor samples, 15 normal samples) at a ratio of 7:3. Clinical data such as gender, age, pathologic stage, Tumor-Node-Metastasis (TNM) stage, treatment type, treatment or therapy, prior malignancy and survival time were also obtained from the TCGA database.

### Analysis of differentially expressed PCGs and lncRNAs

In the training dataset, EdgeR package version 3.8.5 was used to analyze the differentially expressed RNAs between tumor and normal samples, including differentially expressed protein-coding genes (DE-PCGs) and DE-lncRs [26]. The thresholds for DE-PCG and DE-lncR selection were based on a *P*≤0.05 and |logFC | ≥ 1. Hierarchical cluster analysis served to analyze sample similarity.

### Identification of m6A methyltransferase-related lncRNAs

The m6A methyltransferase-related genes were acquired from multiple literatures [27–29]. Four differentially expressed m6A methyltransferase-related genes (DE-m6A methyltransferase genes) were obtained through the intersection analysis with DE-PCGs. The ssGSEA was applied to estimate the m6A methyltransferase score of each sample in the TCGA-HCC training cohort [30,31]. Then, based on the Spearman correlation analysis, lncRNAs related to the m6A methyltransferase, which were highly correlated with the immune score, were identified (|R| > 0.3, *P*<0.05). The prognosis-related lncRNAs were screened by Kaplan–Meier (K–M) survival analyses (*P*<0.2). The intersection of m6A methyltransferase-related and prognosis-related lncRNAs were considered as the candidate m6A methyltransferase-related lncRNAs.

### Establishment and validation of the prognostic gene signature

Univariate Cox regression analysis was used to select factors associated with prognosis. Then, in combination with Cox and Lasso regression analyses, we used R package glmnet to choose the penalty regularization parameter lambda (λ) of the cross-validation routine with an n-fold equal to 10 [32]. Meanwhile, the choice of variables was identified by λ.min. Finally, according to the risk score for each patient, survival analysis, scatter diagram, and heatmap were performed in R software. The time-dependent receiver operating characteristic (ROC) curves were used to evaluate the prognostic prediction accuracy of gene signature and the area under curve (AUC) was measured by package ‘survivalROC’ in R [33]. The prognostic gene signature was verified in the internal validation set. Moreover, univariate and multivariate Cox regression analyses were performed to identify independent prognostic factors.

### Construction of a predictive nomogram

Univariate and multivariate Cox regression analyses were applied to find the independent prognostic factors, which were further visualized via the ‘forestplot’ package in R. Then the nomogram was established by the ‘rms’, ‘nomogramEx’ and ‘regplot’ package in R [34]. Subsequently, calibration curves were plotted to assess the agreement between actual and predicted survival. Furthermore, decision curve analysis (DCA) was used to evaluate the clinical benefits of the nomogram.

### Gene set enrichment analyses

We conducted GSEA to uncover the signaling pathways and biological processes (BPs) in which differentially expressed genes (DEGs) between high- and low-risk subgroups were enriched. A total of 454 DEGs (160 up-regulated and 294 down-regulated) were identified as differentially expressed in high-risk compared with low-risk groups (**Supplementary Figure S2 and Table S1**). GSEA was employed to obtain Gene Ontology (GO) information including BPs, the cellular component (CC) and molecular function (MF). The Kyoto Encyclopedia of Genes and Genomes (KEGG) pathway analysis served to annotate the potential pathways. *P*<0.05 was considered significant and the graph was constructed by the gplots package in R software.

### ESTIMATE algorithm

Stromal and immune scores were evaluated by the ESTIMATE package (version 2.15.3) in R. ESTIMATE is an algorithmic tool that uses the gene expression profiles of 141 immune genes and 141 stromal genes to generate ESTIMATE score to predict tumor purity [30]. The presence of infiltrating immune cells in high- and low-risk groups was calculated with ssGSEA software.

### Statistical analysis

All analyses were performed using R version 3.5.1. Unless otherwise noted, *P*<0.05 was considered to be significant.

## Results

### Identification of DE-PCGs and -lncRNAs in HCC

**Figure 1:**
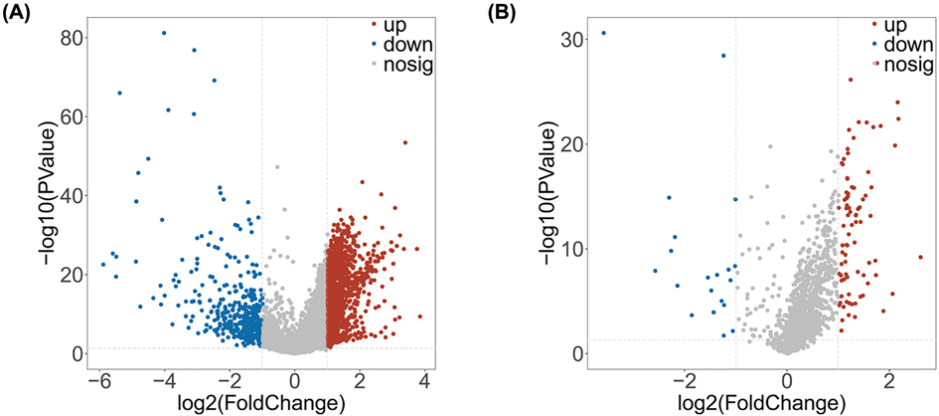
DE-PCGs and DE-lncRs in HCC (**A,B**) Volcano plots of DE-PCGs (A) and DE-lncRs (B) in HCC. The red dots in the plot represent up-regulated genes and blue dots represent down-regulated genes with statistical significance. Gray dots represent no DEGs.

To identify PCGs and lncRNAs that may be involved in the pathogenesis of HCC, the transcription profiles of HCC patients (*n*=260) and healthy subjects (*n*=35) were compared in the training set. After a robust filtering procedure (*P*≤0.05 and |logFC| ≥ 1), 1916 PCGs were significantly modulated (Figure 1A; **Supplementary Table S2**). More than a hundred lncRNAs were detectable, of which 80.6% were up-regulated and 19.4% were down-regulated (Figure 1B; **Supplementary Table S3**). The DE-PCGs and -lncRNAs with similar expression levels were clustered using the systematic cluster analysis (**Supplementary Figure S3**). As displayed in Table 1, the most up-regulated PCG was *AKR1B10* with 3.84-logFC, and the most down-regulated PCG was *HAMP* with −5.90-logFC. Besides, the most up- and down-regulated lncRNAs were *ST8SIA6-AS1* (2.60-logFC) and *LINC01093* (−3.60-logFC), respectively. The expression patterns of the top ten up- and down-regulated PCGs and lncRNAs are illustrated in **Supplementary Figure S3, and Tables S4 and S5**.

**Table 1:** Statistical analysis of all of the differentially expressed PCGs and lncRNAs

DE RNAs	Total number	Number of up-regulated	Number of down-regulated	The most up-regulated (logFC)	The most down-regulated (logFC)
PCG	1916	1500	416	AKR1B10 (3.84)	HAMP (−5.90)
LncRNA	103	83	20	ST8SIA6-AS1 (2.60)	LINC01093 (−3.59)

Abbreviation: DE RNA, differentially expressed RNA.

### Identification of prognostic m6A methyltransferase-related lncRNAs

**Figure 2:**
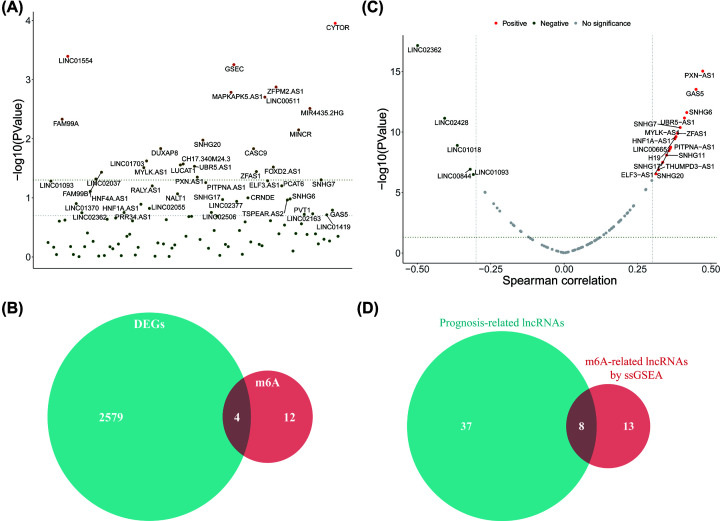
Identification of prognostic m6A methyltransferase-related lncRNAs (**A**) K–M survival analysis of 45 prognosis-related lncRNAs. (**B**) Intersection analysis of m6A methyltransferase genes and DE-PCGs. (**C**) Spearman correlation analysis of m6A methyltransferase scores and DE-lncRs. (**D**) Intersection analysis of prognostic-related lncRNAs and m6A methyltransferase score-related lncRNAs.

Transcription data of 260 patients in the TCGA-HCC training cohort with complete clinical information were used in the present study. K–M survival analyses were used to screen out 45 prognostic-related lncRNAs (Figure 2A and Table 2). Furthermore, the m6A methyltransferase scores in each sample were calculated through ssGSEA based on the reference of the DE-m6A methyltransferase (*METTL3, VIRMA, IGF2BP1* and *IGF2BP2*) gene sets derived from the intersection analysis of 20 m6A methyltransferase genes and DE-PCGs (Figure 2B). After Spearman correlation analyses, 21 lncRNAs were identified as m6A methyltransferase-related lncRNAs (|R| > 0.3, *P*<0.05; Figure 2C). Finally, eight candidate m6A methyltransferase-related lncRNAs (*LINC01093, LINC02362, SNHG20, SNHG17, ZFAS1, SNHG6, SNHG7* and *GAS5*) were obtained by intersection analysis for further research (Figure 2D).

**Table 2:** The 45 prognosis-related lncRNAs

Prognostic-related lncRNA	*P*-value
CASC9	0.0148133
CH17.340M24.3	0.0268725
CRNDE	0.1003839
CYTOR	0.0001119
DUXAP8	0.0147407
ELF3.AS1	0.0517343
FAM99A	0.0046928
FAM99B	0.037008
FOXD2.AS1	0.0302527
GAS5	0.1616622
GSEC	0.0005606
HNF1A.AS1	0.1288273
HNF4A.AS1	0.0779326
LINC00511	0.0019846
LINC01093	0.0522651
LINC01370	0.1255833
LINC01419	0.1944095
LINC01554	0.0004055
LINC01703	0.0238661
LINC02037	0.0481163
LINC02055	0.1514205
LINC02163	0.1866274
LINC02362	0.1794657
LINC02377	0.1152222
LINC02506	0.1772376
LUCAT1	0.0278805
MAPKAPK5.AS1	0.0016472
MINCR	0.007126
MIR4435.2HG	0.0030908
MYLK.AS1	0.0309763
NALT1	0.0858859
PCAT6	0.0628995
PITPNA.AS1	0.0554125
PRR34.AS1	0.1776464
PVT1	0.1908601
PXN.AS1	0.0448655
RALY.AS1	0.0630094
SNHG17	0.1073125
SNHG20	0.0105894
SNHG6	0.1046746
SNHG7	0.049813
TSPEAR.AS2	0.1091516
UBR5.AS1	0.0293862
ZFAS1	0.0361043
ZFPM2.AS1	0.0013408

### Establishment of m6A methyltransferase-related lncRNAs signature

**Figure 3:**
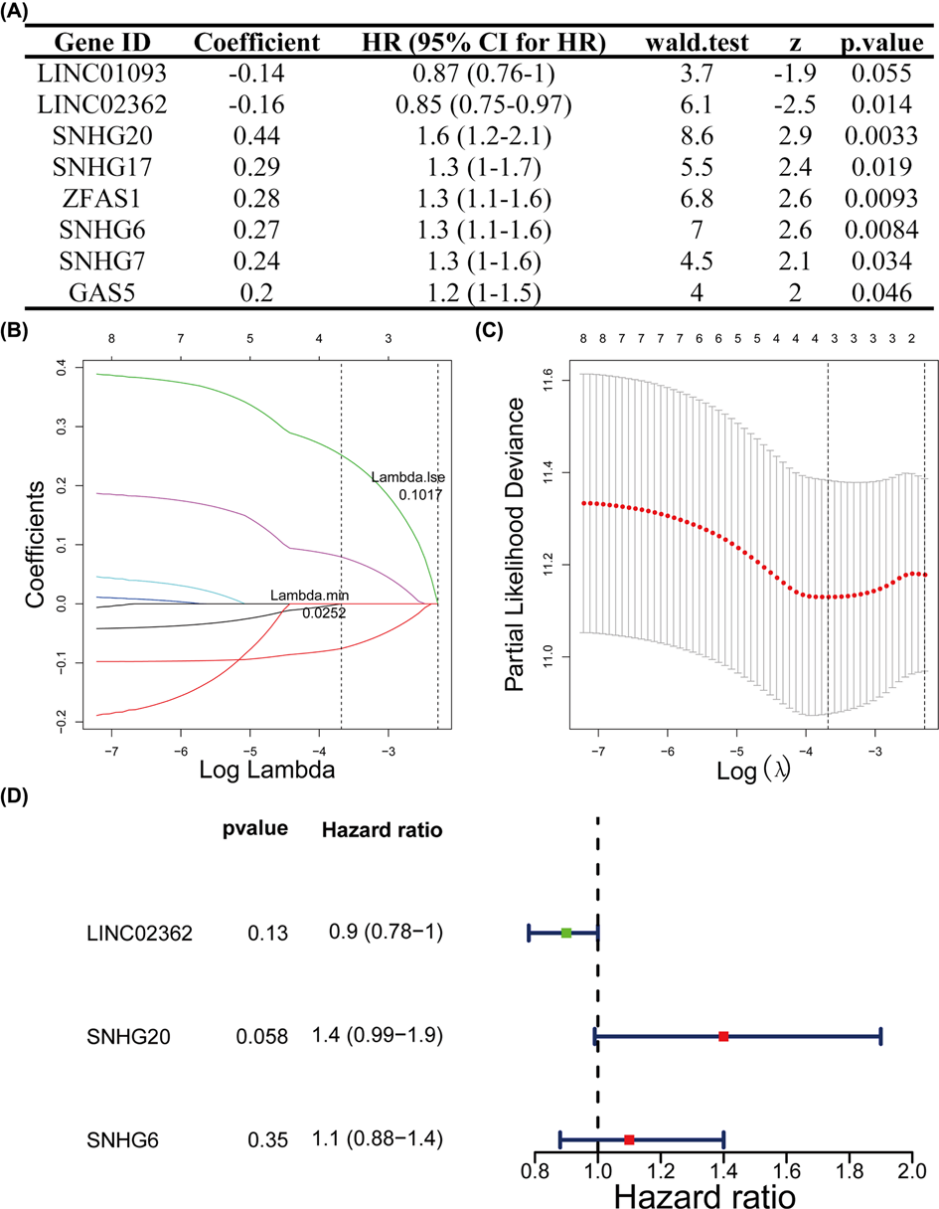
Establishment of m6A methyltransferase-related lncRNAs signature (**A**) The univariate Cox regression analysis of eight m6A methyltransferase-related lncRNAs. (**B–D**) LASSO regression was performed, calculating the minimum criteria (B,C) and forest plot summary of the HR values of the prognostic lncRNAs (D).

The univariate Cox regression analysis demonstrated that *LINC01093* (*P*=0.055) and *LINC02362* (*P*=0.014) were protective genes with hazard ratio (HR) less than 1, whereas *SNHG20* (*P*=0.0033), *SNHG17* (*P*=0.019), *ZFAS1* (*P*=0.0093), *SNHG6* (*P*=0.0084), *SNHG7* (*P*=0.034) and *GAS5* (*P*=0.046) were risky genes with HR greater than 1 (Figure 3A and Table 3). Following this, eight significant m6A methyltransferase-related lncRNAs (*P*<0.2) were subjected to LASSO modeling (Figure 3B,C). Then, three m6A methyltransferase-related lncRNAs were selected based on λ.min values, and the HR values of the three candidate lncRNAs were calculated (Figure 3D and **Supplementary Table S6**).

**Table 3:** The univariate Cox regression analysis of eight m6A methyltransferase-related lncRNAs

Gene ID	Coefficient	HR (95% CI for HR)	Wald test	z	*P*.value
LINC01093	−0.14	0.87 (0.76–1)	3.7	−1.9	0.055
LINC02362	−0.16	0.85 (0.75–0.97)	6.1	−2.5	0.014
SNHG20	0.44	1.6 (1.2–2.1)	8.6	2.9	0.0033
SNHG17	0.29	1.3 (1–1.7)	5.5	2.4	0.019
ZFAS1	0.28	1.3 (1.1–1.6)	6.8	2.6	0.0093
SNHG6	0.27	1.3 (1.1–1.6)	7	2.6	0.0084
SNHG7	0.24	1.3 (1–1.6)	4.5	2.1	0.034
GAS5	0.2	1.2 (1–1.5)	4	2	0.046

**Figure 4:**
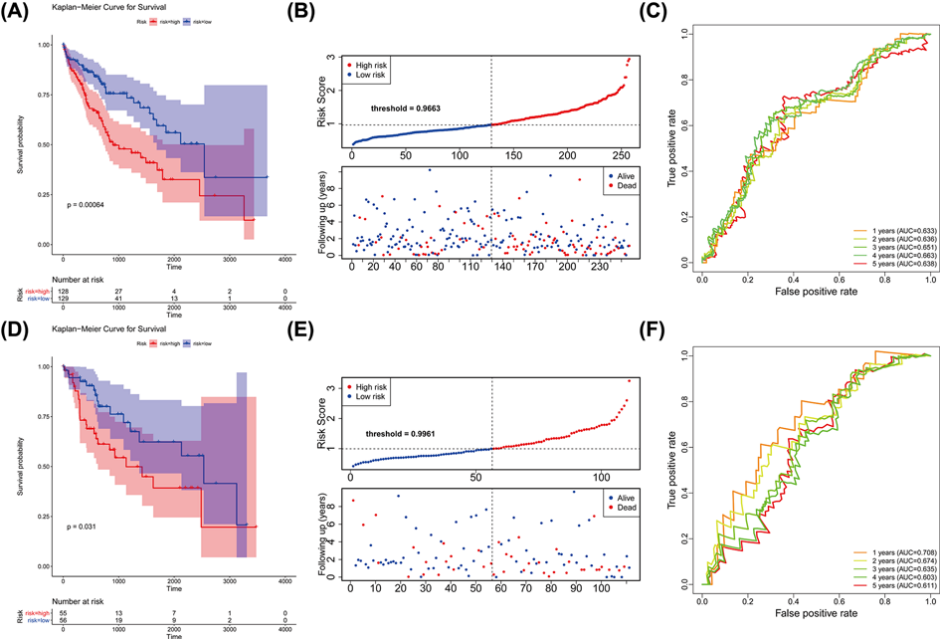
m6A methyltransferase-related lncRNA signature was a prognostic biomarker for OS in the TCGA-HCC cohort (**A–F**) K–M survival, risk score and time-dependent ROC curves of OS according to m6A methyltransferase-related lncRNA signature groups in TCGA-HCC training and testing cohorts. The entire cohort was divided into the training and testing cohorts at the 7:3 cutoff. The cohorts were all stratified at a median cutoff of the risk-scores to form high-risk and low-risk groups. The AUC was assessed at 1, 2, 3, 4 and 5 years.

We classified patients into low-risk or high-risk groups based on the median risk score. K–M survival curve indicated that the low-risk patients in the training cohort had a better OS than high-risk patients (*P*=0.00064) (Figure 4A,B). Time-dependent ROC analysis showed that the AUC of the risk score predicted OS was 0.633 at 1 year, 0.636 at 2 years, 0.651 at 3 years, 0.663 at 4 years and 0.638 at 5 years in the training cohort (Figure 4C). Then, the prognostic model was further validated in the testing set, and consistently, a high-risk score was associated with a worse prognosis (Figure 4D,E). In the testing cohort, the significant prognostic value was *P*=0.031 and AUC with 1-, 2-, 3-, 4- and 5 years were 0.708, 0.674, 0.635, 0.603 and 0.611, respectively (Figure 4F). Also, the PCA analysis indicated that there was a significant disparity in patients between high- and low-risk groups (**Supplementary Figure S4**).

### Prognostic risk score displayed strong correlations with clinicopathological features and survival in HCC patients

**Figure 5:**
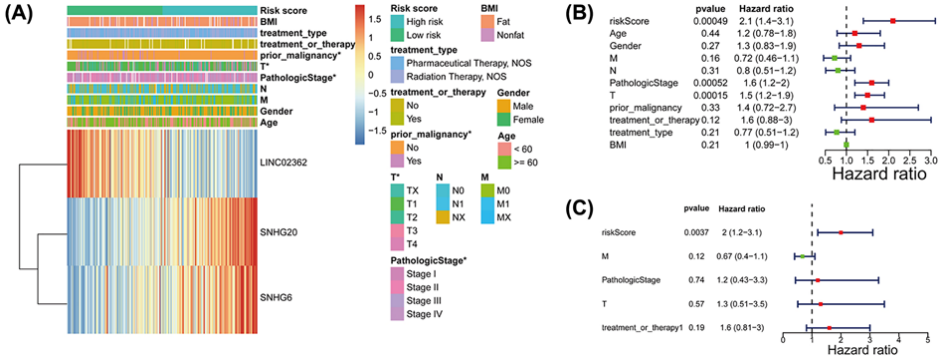
Relationship between the risk scores and clinicopathological factors in the training cohort (**A**) The heatmap showed the distribution of clinicopathological factors and three m6A methyltransferase-related lncRNAs between the high- and low- risk groups. m6A methyltransferase-related lncRNA signature is an independent prognosis factor in the nomogram. (**B,C**) Forest plot summary of the univariate (B) and multivariable (C) Cox analyses of the m6A methyltransferase-related lncRNA signature and clinicopathological characteristics.

We used the median risk score as a cutoff to define the groups of HCC patients with high and low scores. The heatmap showed the expression of the three m6A methyltransferase-related lncRNAs in high- and low-risk patients of the training cohort. Significant differences between the high-risk and low-risk groups of patients concerning prior malignancy (*P*<0.05), T stage (*P*<0.05) and pathologic stage (*P*<0.05) were identified (Figure 5A). We then observed that the risk scores were significantly different between patients categorized by prior malignancy, T stage and pathologic stage (**Supplementary Figure S5**). These results revealed that risk scores were positively associated with the T stage and pathologic stage, and the ‘no prior malignancy’ subgroup exhibiting the worst prognosis had the highest risk score. Stratification survival analyses showed that the risk score could efficiently predict the OS in most subgroups based on different clinical characteristics (**Supplementary Figure S6**). Similar procedures were performed in the testing set, and the results were almost consistent with the training set (**Supplementary Figures S7 and S8**).

Based on the univariate and multivariate Cox proportional hazards regression analyses, only one independent predictor (risk score) was identified in the HCC. Univariate Cox proportional hazards regression analysis demonstrated that age (*P*=0.44), gender (*P*=0.27), N stage (*P* = 0.31), prior malignancy (*P*=0.33), treatment type (*P*=0.21) and BMI (*P*=0.21) had no impact upon OS (Figure 5B and **Supplementary Table S7**). Multivariate Cox proportional hazards regression analysis showed a significant correlation between risk score (HR = 2, *P*=0.0037) and OS in HCC patients (Figure 5C and **Supplementary Table S8**). Then, the predictor was incorporated to build the nomogram.

Based on the nomogram, each patient will obtain a corresponding score from each prognostic parameter to estimate the 3- and 5-year survival rates. Patients with a higher total score represented a higher mortality rate. We concluded that the risk score played an important role in patient prognosis. Moreover, calibration curves revealed that our model had good prediction accuracy. The DCA demonstrated that the nomogram not only had the advantage of the risk score but also a great potential for clinical application (**Supplementary Figure S9**).

### Functional enrichment analyses of the DEGs between the high- and low-risk groups

**Figure 6:**
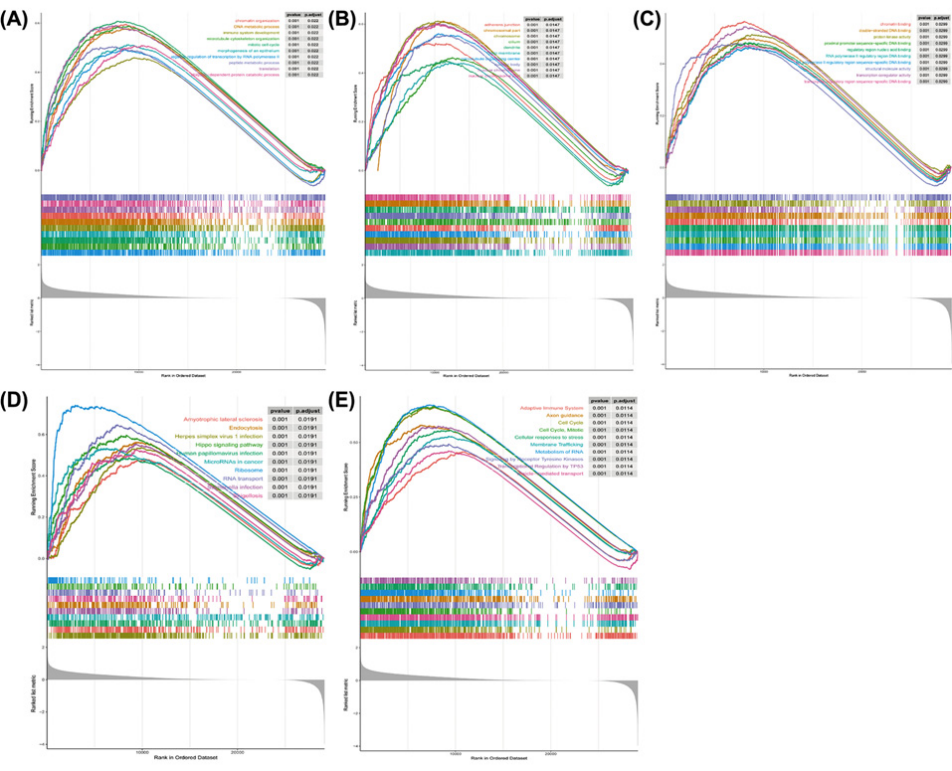
Functional enrichment analyses of the DEGs between the high- and low-risk groups (**A–C**) The top ten GO of GSEA, including BPs (A), CC (B) and MFs (C). (**D**) KEGG pathway analysis. (**E**) Reactome pathway analysis.

To explore the underlying molecular mechanisms of the signature, we conducted GSEA to compare 454 DEGs between high- and low-risk groups (risk DEGs) in 371 TCGA-HCC patients of the whole set. We enriched the functions of these mRNA (*P*<0.05). As shown in Figure 6A, ‘negative regulation of transcription by RNA polymerase II’ and ‘microtubule cytoskeleton organization’ were significantly enriched for BPs (**Supplementary Table S9**), while for the CC was ‘Golgi membrane’, for MFs were ‘transcription regulatory region sequence-specific DNA binding’ and ‘RNA polymerase II regulatory region sequence-specific DNA binding’ (Figure 6B,C and **Supplementary Tables S10 and S11**). KEGG pathway analysis revealed that the DEGs participated in ‘Herpes simplex virus 1 infection’, ‘Amyotrophic lateral sclerosis’, ‘Human papillomavirus infection’ and ‘MicroRNAs in cancer’ (Figure 6D and **Supplementary Table S12**). Furthermore, the DEGs participating in the ‘Axon guidance’ pathway indicated their involvement in the neurological invasion of HCC (Figure 6E and **Supplementary Table S13**). Moreover, the GSEA also demonstrated that the terms/pathways of immune response and immune system process were highly enriched in the risk DEGs (**Supplementary Table S14**). All these demonstrated that the risk score could be related to the tumor immune microenvironment, which was so important for HCC.

### The landscape of TME immune cells infiltration in the two risk subgroups

The tumor immune microenvironment is considered the ‘seventh largest representative characteristic’ of tumor and is composed of innate immune cells, adaptive immune cells, cytokines and cell surface molecules. These immune microenvironment components constitute a complex regulatory network and play a pivotal role in tumorigenesis and development [35,36]. Considering that the risk score also had a close connection with immune reactions, we investigated the difference in immune signatures between the two risk subgroups.

**Figure 7:**
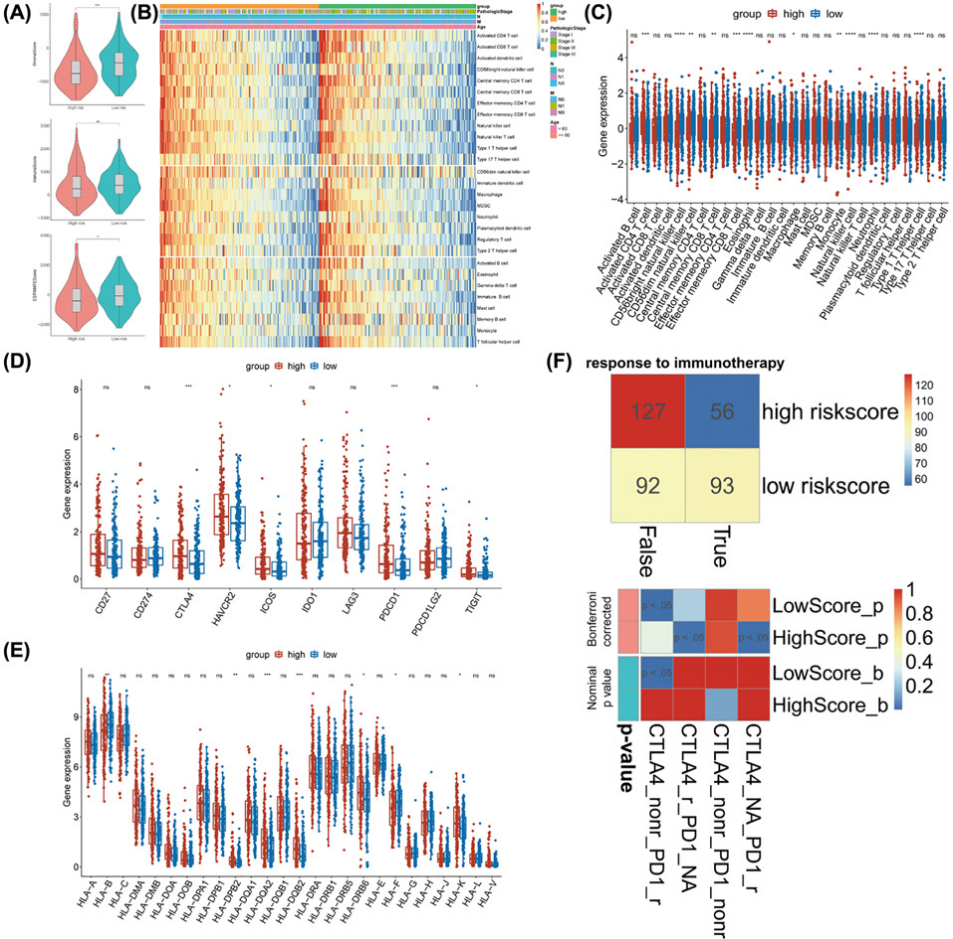
The landscape of TME immune cells infiltration and evaluation of therapeutic response in the two risk subgroups (**A**) StromalScore, ImmuneScore and ESTIMATEScore in the two risk subgroups. **P<0.01, ****P<0.0001. (**B,C**). The ratio of pro-tumor signatures and anti-tumor signatures was significantly increased in the low-risk subgroup. (**D,E**). Almost all immune-checkpoints and *HLA* expression were up-regulated in the high-risk subgroup. (**F**) TIDE algorithm showed differential immunotherapeutic response in the two risk subgroups. Abbreviations: HLA, human leukocyte antigen; TIDE, Tumor Immune Dysfunction and Exclusion. *P<0.05, ***P<0.001.

Based on the ImmuneScore, StromalScore and ESTIMATEScore generated by the ESTIMATE algorithm, as shown in Figure 7A, the stromal score and ESTIMATE score were significantly negatively correlated with the risk score (*P*<0.01). ssGSEA analysis revealed that the ratio of pro-tumor signatures (CD56bright natural killer cell, central memory CD8 T cell, effector memory CD8 T cell, natural killer cell, type 1 T helper cell) and anti-tumor signatures (CD56dim natural killer cell, macrophage, neutrophil) were significantly elevated in the low-risk subgroup, indicating that the low-risk subgroup was interrelated with increased immune/inflammation activity. On the other hand, the high-risk subgroup had relatively higher activated CD4 T cells (Figure 7B,C).

### Low-risk patients were likely to be more sensitive to immunotherapy

Pioneering investigations revealed that immunotherapy targeting immune-checkpoints and the human leukocyte antigen (HLA) brought great hope for the clinical treatment of human cancers [37]. We investigated the association between the risk score and several well-known immune checkpoints and HLA expression levels in the TCGA database. Then we discovered that the expression of almost all immune checkpoints (*CTLA4, HAVCR2, ICOS, PDCD1* and *TIGIT*) and HLA (*HLA-DPB2, HLA-DQA2, HLA-DQB2, HLA-DRB6* and *HLA-K*) were up-regulated in the high-risk group, which indicated the overexpression of immune checkpoints might be the reason for the poor prognosis (Figure 7D,E). Here, patients in the high-risk group may be more suitable for immunotherapy. Then, we used the Tumor Immune Dysfunction and Exclusion (TIDE) algorithm to predict the likelihood of the risk model for immunotherapy. Interestingly, patients in the low-risk group were more likely to respond to immunotherapy than those in the high-risk group (Figure 7F). These data further confirmed that the low-risk group had a better prognosis and might have a better treatment prospect for the immunotherapy.

## Discussion

Currently, there is lack of effective clinical interventions in HCC, which lead to the high metastasis rate and mortality rate. Thus, it is critical to understand the molecular mechanisms underlying HCC development. There is growing evidence that m6A modification participates in the development and progression of HCC [13–18]. *METTL3* increases the expression of HCC-derived growth factor (HDGF) by up-regulating *LINC00958*, thereby promoting HCC progression and lipogenesis [38]. Lan et al. demonstrated that *KIAA1429* promotes the progression of HCC by modifying *lncRNA GATA3* with m6A [39]. However, studies on m6A-related lncRNAs in the prognosis of HCC were limited. Therefore, we focused on their interactions to identify potential prognostic biomarkers or therapeutic targets for HCC.

We identified 21 m6A-related lncRNAs from 260 HCC patients, 3 of which were selected into the m6A-related lncRNA prognostic signature (*LINC02362, SNHG20* and *SNHG6*). *LINC02362* was included in a prognostic model for HCC, which was significantly correlated with tumor grade, stage and T stage (Zhao et al., 2020) [56]. Tu et al. reported that HBV x protein promoted the proliferation of HCC cell through the lncRNA *SNHG20/PTEN* signaling pathway [40]. According to reports, up-regulated *SNHG6* activates the expression of *SERPINH1* by competitively binding *miR-139-5p*, thereby promoting the development of HCC [41]. These three lncRNAs have been reported to be associated with HCC, but their interactions with m6A-related genes have not been studied yet. Our results contribute to identify lncRNAs related to m6A modulators, and thus revealing their potential roles in the oncogenesis and progression of HCC.

Our study showed that the lncRNA prognostic signature was closely related to the TME, which was correlated with the prognosis of HCC patients. It has been reported that several components of the TME, including immune cells, cytokines, chemokines, inhibitory receptors and ligands, are critical factors in tumorigenesis and progression [42,43]. We further found that almost all immune checkpoints and HLA expression were significantly up-regulated in high-risk patients, suggesting that poor prognosis might be related to the induction of high immune checkpoint expression. In HCC, it is well known that the increase in the number of *PD-1*^+^ and CD8^+^ T cells in tumor tissues and circulation indicates a high recurrence rate and poor prognosis after surgery [44]. Research also showed that the up-regulation of *PD-L1* in HCC cells was induced by a variety of cytokines, especially *IFN-γ*, which in turn impaired anti-tumor immunity and promoted apoptosis of CD8^+^ T cells [45]. Although the numbers of NK cells and CD8^+^ T cells were accumulated during tumor progression, the activity of which will be greatly inhibited after binding with *PD-Ls*, thus weakening the anti-tumor effect of these cells [46]. On the other hand, our result showed that the ratio of pro-tumor signatures and anti-tumor signatures was significantly elevated in low-risk patients, indicating that better prognosis might be attributed to the increased immune/inflammation activity. Previous studies have demonstrated that increased infiltrations of NK, T and NKT cells are positive prognostic factors for HCC [47–49]. In addition, Xu et al. also showed that a higher immunoreactive environment could inhibit tumor growth, progression, invasion and metastasis [50].

Currently, immunotherapy has been applied to the treatment of various cancers and changed the landscape of cancer care. For example, blocking the combination of *PD-1* and *PD-L1* can rescue the function of effector T cells to promote their function of killing tumor cells [51]. The expression of *PD-L1* in tumors is the main factor in determining whether a patient is eligible for *PD-1/PD-L1* axis immunotherapy. In clinical practice, however, quite a few *PD-L1* positive patients respond poorly to the *PD-1/PD-L1* axis treatment, while some patients with negative *PD-L1* have a surprising response to treatment [52–55]. Consistently, our study observed that patients in the high-risk group with up-regulation of immune-checkpoints had a weaker response to immunotherapy. On the contrary, patients in the low-risk subgroup with higher immune/inflammation activity were more likely to benefit from immunotherapy, indicating that immune cells infiltration might be a predictor of immunotherapy response.

There were still limitations in our study. Firstly, our results were only based on the TCGA database without external validation. Secondly, no experiment was used to confirm the interaction between the prognostic lncRNAs and m6A modulators in HCC. However, we will conduct further research to provide a better understanding of the above results. In conclusion, we established a novel prognostic signature for HCC based on m6A-related lncRNAs. Meanwhile, the present study also deepened the understanding of the immune microenvironment status of HCC, and laid a theoretical foundation for the prediction of immunotherapy for HCC patients.

## Supplementary Material

Supplementary Figures S1-S9

Supplementary Tables S1-S14

## Data Availability

The data used to support the findings of the present study are available from the corresponding author upon request.

## Abbreviations

AUC: area under curve
BP: biological process
CC: cellular component
DE-lncR: differentially expressed long non-coding RNA
DE-PCG: differentially expressed protein-coding gene
DEG: differentially expressed gene
HBV: hepatitis B virus
HCC: hepatocellular carcinoma
HLA: human leukocyte antigen
HR: hazard ratio
KEGG: Kyoto Encyclopedia of Genes and Genomes
K–M: Kaplan–Meier
lncRNA: long non-coding RNA
MF: molecular function
miRNA: microRNA
mRNA: messenger RNA
m6A: N6-methyladenosine
ROC: receiver operating characteristic
TCGA: The Cancer Genome Atlas
TME: tumor microenvironment
ssGSEA: single sample Gene Set Enrichment Analysis
