# Supplementary material for: Identification of m6A methyltransferase-related lncRNA signature for predicting immunotherapy and prognosis in patients with hepatocellular carcinoma
Source: Biosci Rep. 2021 Jun 8;41(6):BSR20210760. doi: 10.1042/BSR20210760 (PMC8188173; doi:10.1042/BSR20210760)
Supplement: Supplementary Figures S1-S9 [file BSR-2021-0760_supp.pdf]

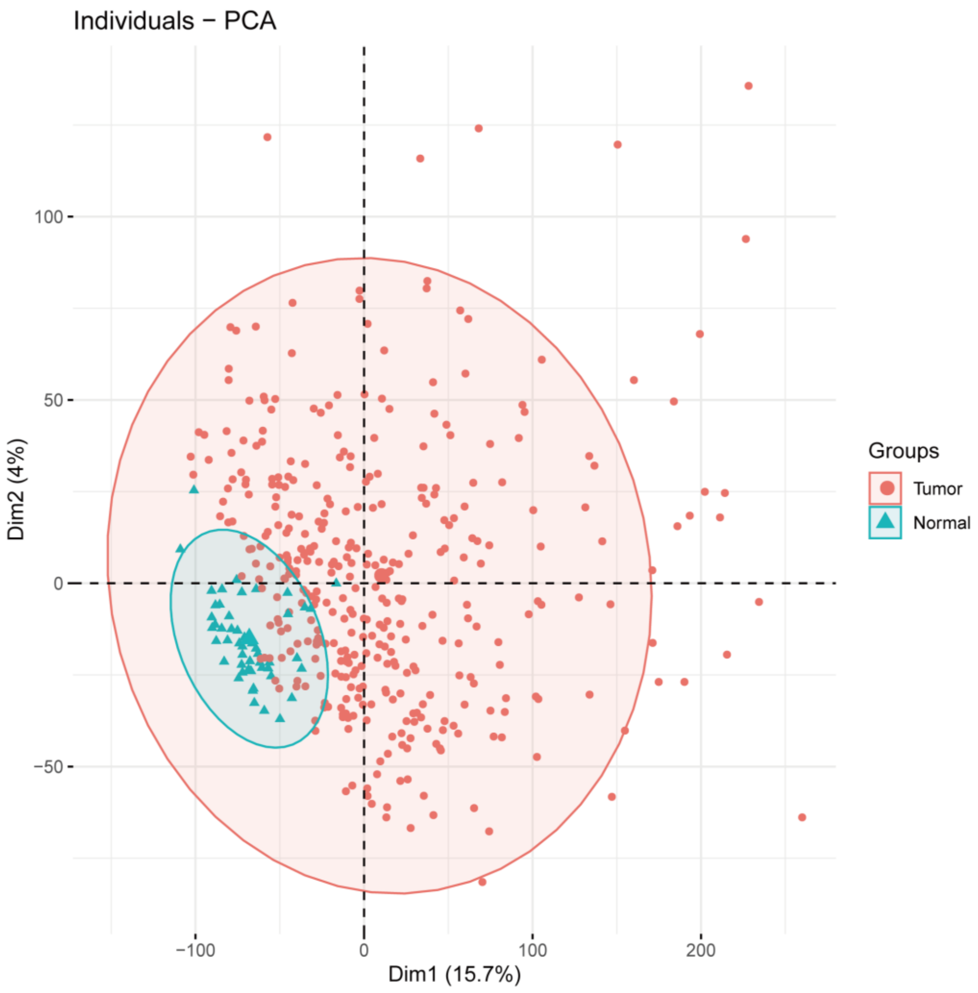

Supplementary Figure 1 legend: Sample quality control chart.

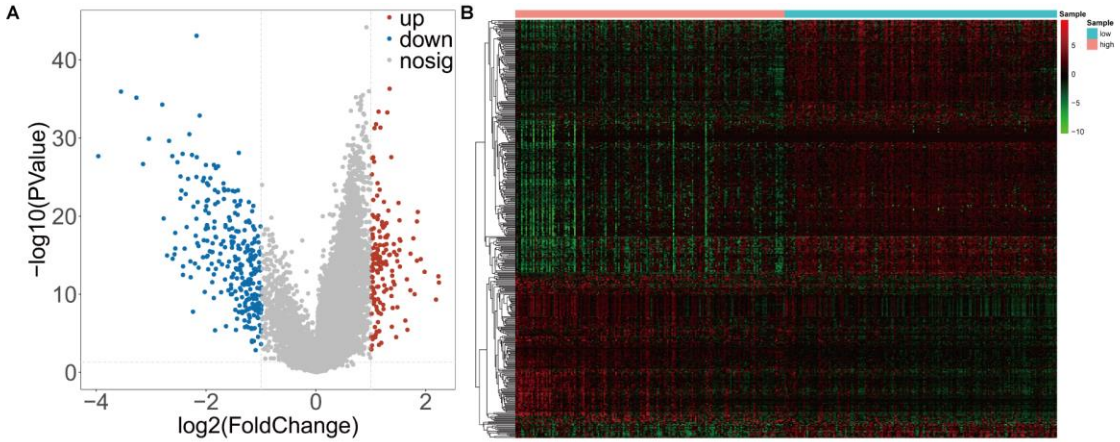

Supplementary Figure 2 legend: Differentially expressed genes (DEGs) in the two risk subgroups. (A). Volcano plots of DEGs. The red dots in the plot represent upregulated genes and blue dots represent downregulated genes with statistical significance. Grey dots represent no differentially expressed genes. (B). Heatmap of significantly DEGs. Red represents higher expression while green represents lower expression.

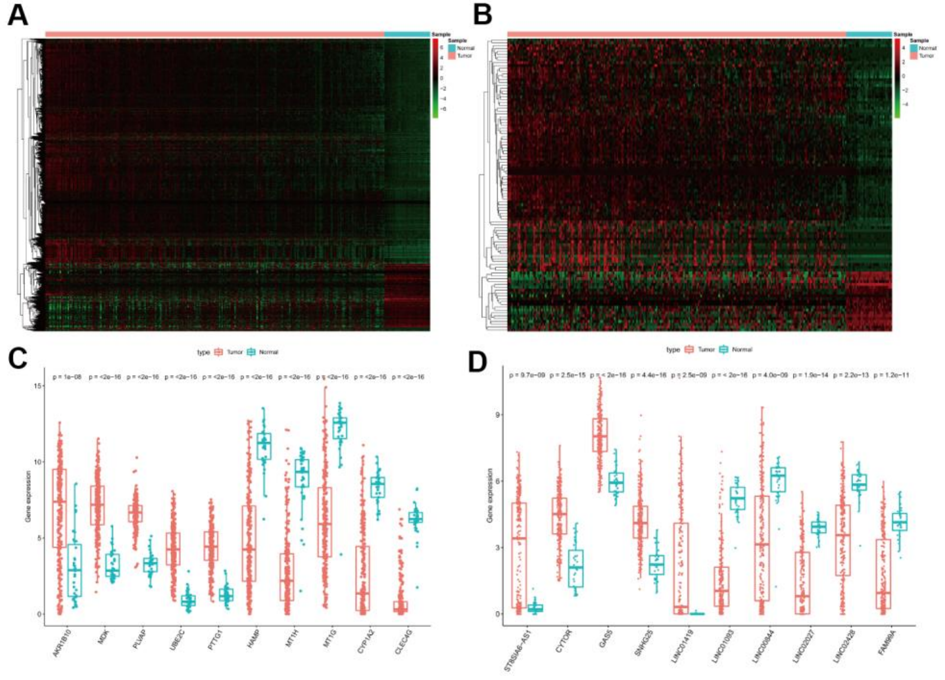

Supplementary Figure 3 legend: DE-PCGs and DE-lncRNAs in HCC. (A, B). Heatmap of significantly DE-PCGs (A) and DE-lncRNAs (B) in HCC. Red represents higher expression while green represents lower expression. (C, D). Expression patterns of the top 10 DE-PCGs (C) and DE-lncRNAs (D) in HCC and normal tissues.

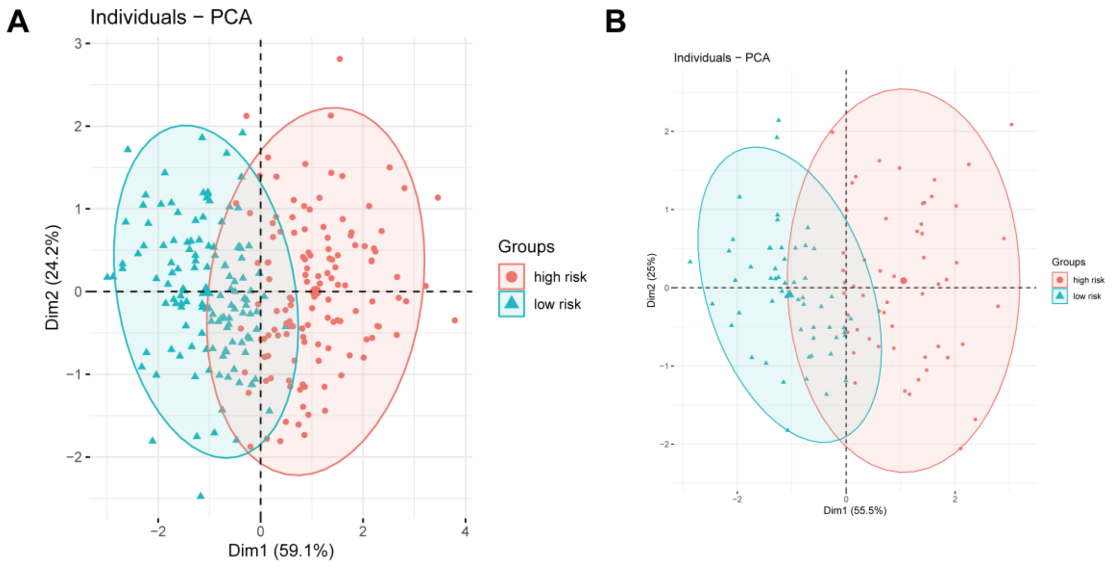

Supplementary Figure 4 legend: PCA analysis in the two risk subgroups. (A). PCA analysis in the training group. (B). PCA analysis in the internal validation set the testing group.

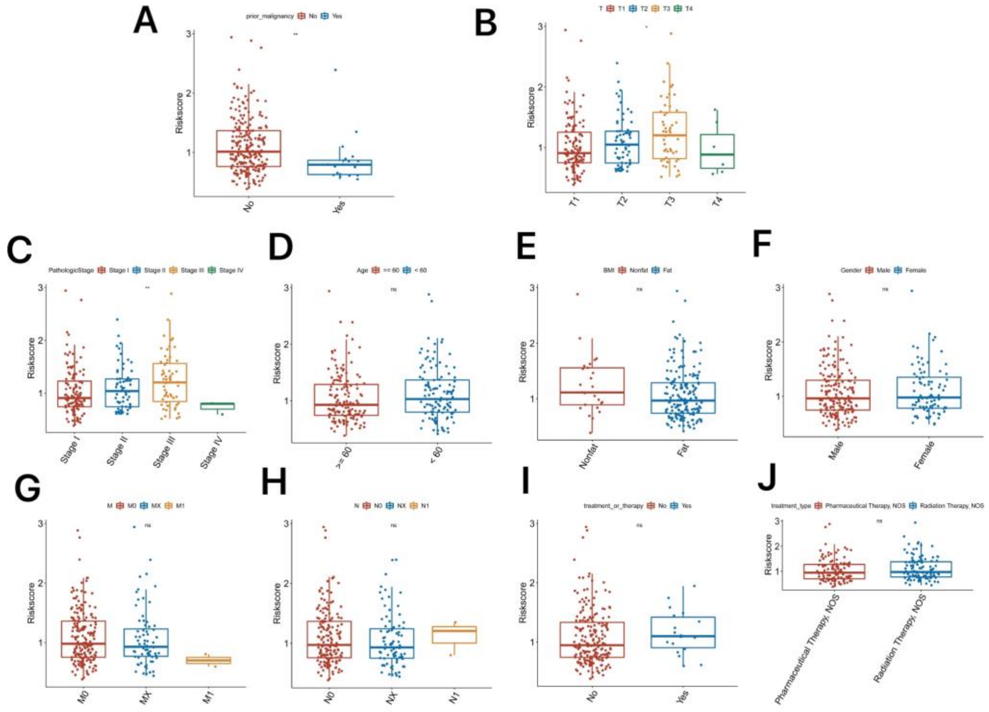

Supplementary Figure 5 legend: Relationship between the risk scores and clinicopathological factors in the training cohort. (A-J). Distribution of risk scores stratified by prior malignancy (A), T stage (B), pathologic stage (C), age (D), BMI (E), gender (F), M stage (G), N stage (H), treatment or therapy (I) and treatment type (J). *P < 0.05, **P < 0.01.

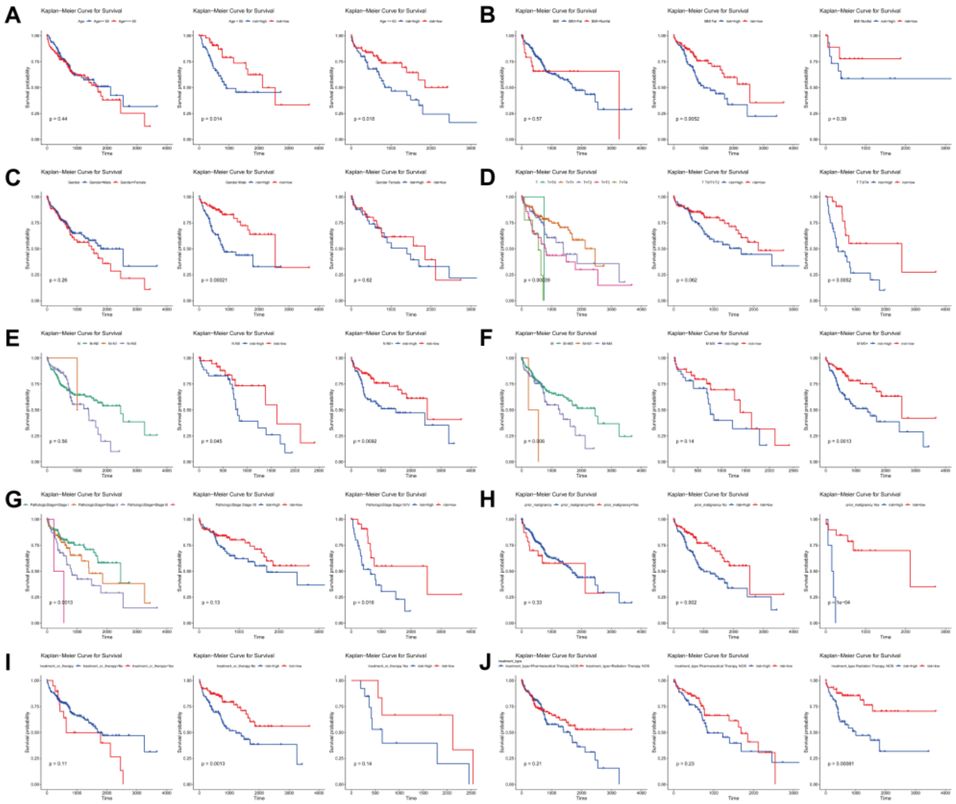

Supplementary Figure 6 legend: Kaplan–Meier curves of clinicopathological characteristics and risk group stratification within the training set for OS. Clinicopathological features included age (A), BMI (B), gender (C), T stage (D), N stage (E), M stage (F), pathologic stage (G), prior malignancy (H), treatment or therapy (I), and treatment type (J).

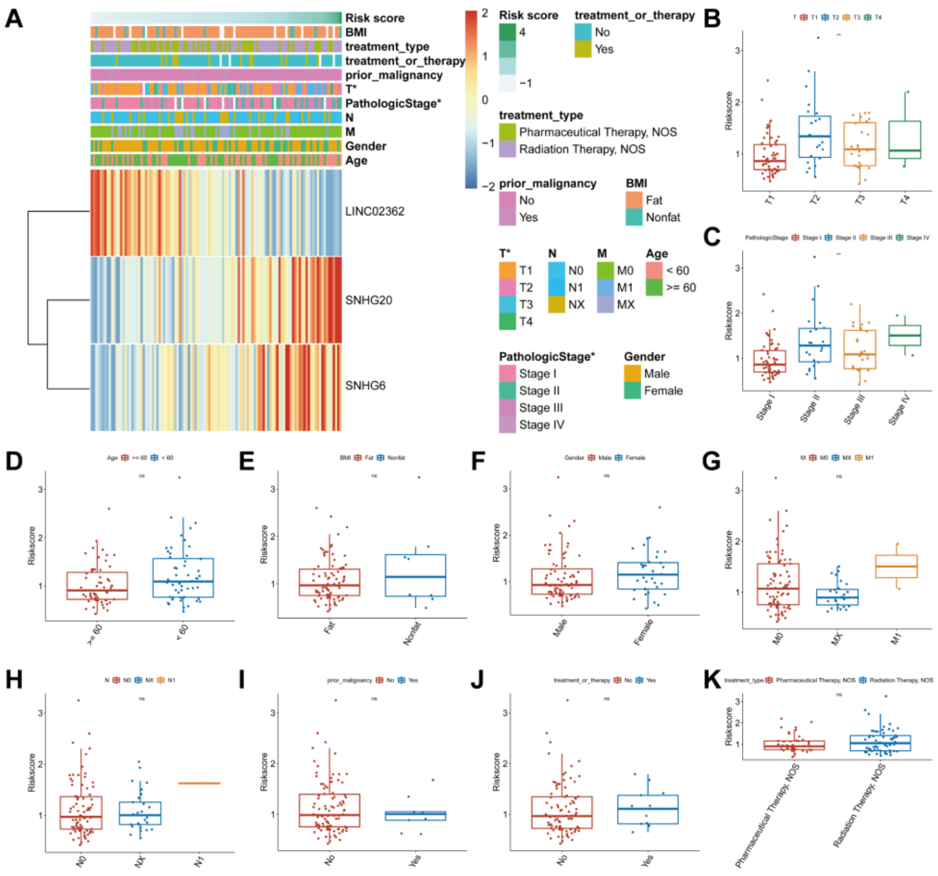

Supplementary Figure 7 legend: Relationship between the risk scores and clinicopathological factors in the testing cohort. (A). The heatmap showed the distribution of clinicopathological factors and three m6A methyltransferase-related lncRNAs between the high- and low- risk groups. (B-K). Distribution of risk scores stratified by T stage (B), pathologic stage (C), age (D), BMI (E), gender (F), M stage (G), N stage (H), prior malignancy (I), treatment or therapy (J) and treatment type (K). *P < 0.05, **P < 0.01.

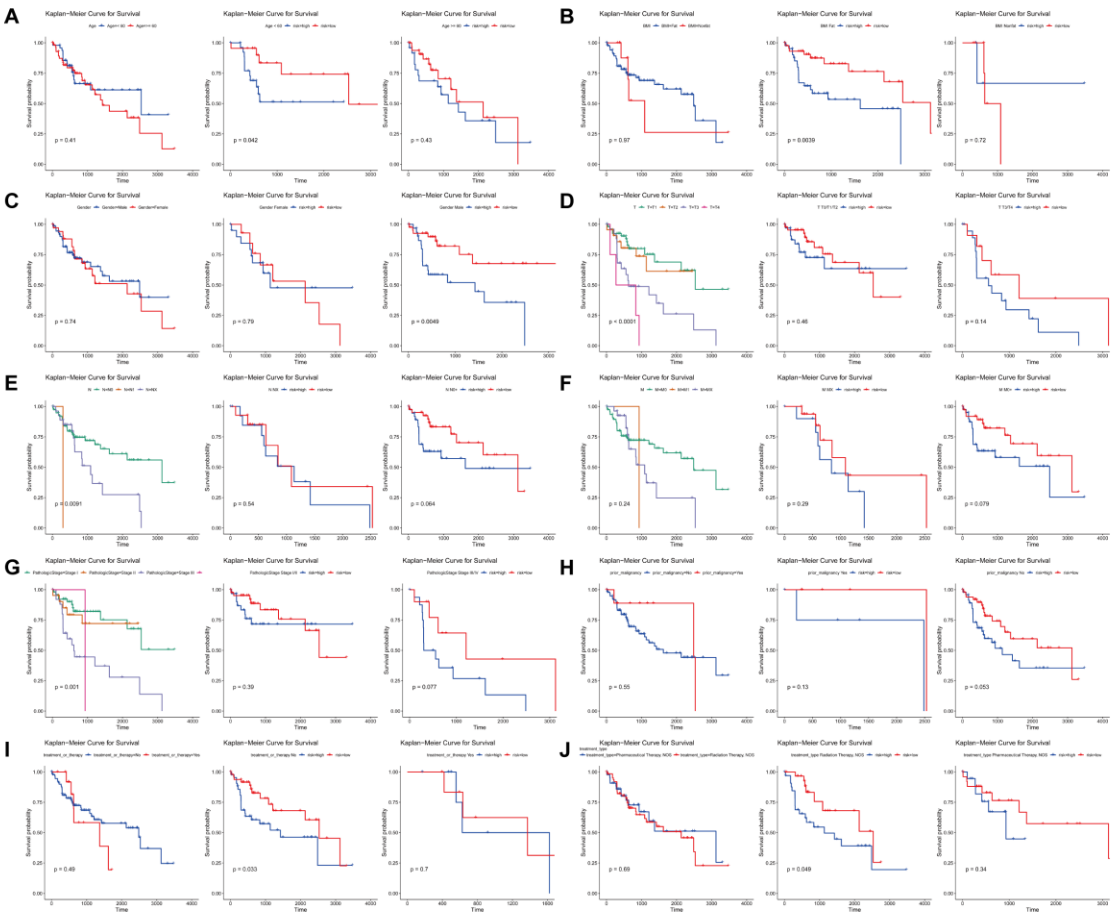

Supplementary Figure 8 legend: Kaplan–Meier curves of clinicopathological characteristics and risk group stratification within the testing set for OS. Clinicopathological features included age (A), BMI (B), gender (C), T stage (D), N stage (E), M stage (F), pathologic stage (G), prior malignancy (H), treatment or therapy (I), and treatment type (J)

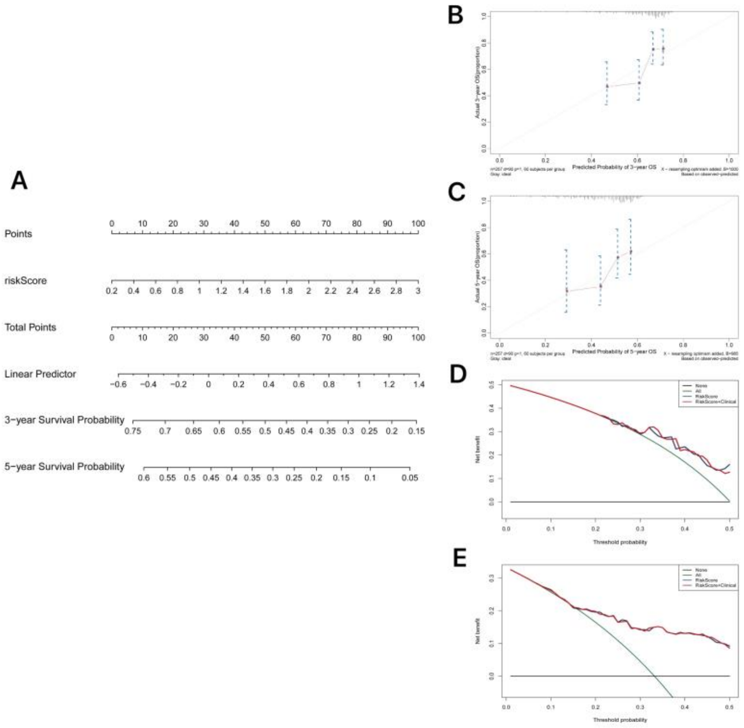

Supplementary Figure 9 legend: m6A methyltransferase-related lncRNA signature is an independent prognosis factor in the nomogram. (A) Nomogram integrating the m6A methyltransferase-related lncRNA signature for predicting the probability of patient mortality at 3- or 5-year OS. (B, C) Calibration curves of the nomogram for predicting the survival outcomes at 3-, and 5-years. The 45-degree line represents the ideal prediction. (D, E) DCA curves of the nomogram for 3-year and 5-year OS.
